# Circadian Oscillations in the Murine Preoptic Area Are Reset by Temperature, but Not Light

**DOI:** 10.3389/fphys.2022.934591

**Published:** 2022-07-22

**Authors:** Nicolás M. Díaz, Shannon A. Gordon, Richard A. Lang, Ethan D. Buhr

**Affiliations:** ^1^ Department of Ophthalmology, University of Washington School of Medicine, Seattle, WA, United States; ^2^ Science of Light Center, Cincinnati Children’s Hospital Medical Center, Cincinnati, OH, United States; ^3^ The Visual Systems Group, Abrahamson Pediatric Eye Institute, Cincinnati Children’s Hospital Medical Center, Cincinnati, OH, United States; ^4^ Division of Pediatric Ophthalmology, Cincinnati Children’s Hospital Medical Center, Cincinnati, OH, United States; ^5^ Department of Ophthalmology, University of Cincinnati College of Medicine, Cincinnati, OH, United States

**Keywords:** preoptic area, circadian rhythm, Opn5, neuropsin, temperature

## Abstract

Mammals maintain their internal body temperature within a physiologically optimal range. This involves the regulation of core body temperature in response to changing environmental temperatures and a natural circadian oscillation of internal temperatures. The preoptic area (POA) of the hypothalamus coordinates body temperature by responding to both external temperature cues and internal brain temperature. Here we describe an autonomous circadian clock system in the murine ventromedial POA (VMPO) in close proximity to cells which express the atypical violet-light sensitive opsin, Opn5. We analyzed the light-sensitivity and thermal-sensitivity of the VMPO circadian clocks *ex vivo*. The phase of the VMPO circadian oscillations was not influenced by light. However, the VMPO clocks were reset by temperature changes within the physiological internal temperature range. This thermal-sensitivity of the VMPO circadian clock did not require functional Opn5 expression or a functional circadian clock within the Opn5-expressing cells. The presence of temperature-sensitive circadian clocks in the VMPO provides an advancement in the understanding of mechanisms involved in the dynamic regulation of core body temperature.

## Introduction

The preoptic area (POA) of the anterior hypothalamus is most commonly associated with its role in the regulation of body temperature and sleep (Tan and Knight, 2018). Increases in environmental warmth activate neurons within the ventromedial POA (VMPO), whose neural activity is correlated with behavioral and physiological mechanisms of cooling (Knox et al., 1973; Bachtell et al., 2003; Tan et al., 2016). In addition to responding to environmental temperature, the VMPO is active in regulation of body temperature changes during sleep (Rothhaas and Chung, 2021). Interestingly, this regulation of body temperature during sleep is independent of the regulation of the circadian rhythm of core body temperature by the brain’s central circadian clock, the suprachiasmatic nucleus (SCN). Lesioning the VMPO actually increases the amplitude of the circadian rhythm of body temperature, suggesting a modulatory role of the VMPO on the range of normal core body temperatures over a circadian cycle (Osborne and Refinetti, 1995; Lu et al., 2000). Such lesions also diminish animals’ ability to maintain set points of body temperature in response to environmental temperature challenges (Teague and Ranson, 1936). Inversely, lesioning of the SCN abolishes the circadian component of body temperature, but leaves the relation between sleep and body temperature intact (Baker et al., 2005). Although not directly connected, the VMPO receives afferent input from the SCN *via* the paraventricular nucleus (PVN) (Vujovic et al., 2015).

It has long been appreciated that neurons in the VMPO are activated by either temperature information relayed by dermal thermosensors or to local changes in brain temperature (Nakayama et al., 1961; Boulant and Hardy, 1974). Local heating of the POA causes systemic body temperature reduction (Magoun et al., 1938; ANDERSSON et al., 1956; Carlisle and Laudenslager, 1979). We have recently identified neurons in the POA which are directly sensitive to violet light and whose activation similarly causes a reduction in core and brown adipose tissue (BAT) temperature (Zhang et al., 2020). These neurons express the atypical opsin, Opn5 (or “neuropsin”), which has been found in both the murine and primate VMPO (Tarttelin et al., 2003; Yamashita et al., 2014). Unlike wild-type mice, *Opn5*-null mice do not regulate their body temperature in response to violet light (Zhang et al., 2020).

Opn5 absorbs UV/violet light ( ∼ 380–400 nm) and acts as a bistable photopigment which retains its retinaldehyde chromophore through successive activation events (Yamashita et al., 2010; Yamashita et al., 2014). In birds, Opn5 is expressed in hypothalamic neurons bordering the third ventricle which act as deep brain photoreceptors regulating seasonal reproduction (Nakane et al., 2010; Nakane et al., 2014). In mammals, Opn5 has been detected in neural tissues such as the brain and retina, as well as non-neural tissues like the gonads, skin, and cornea (Tarttelin et al., 2003; Kojima et al., 2011; Buhr et al., 2015; Díaz et al., 2020). During retinal development Opn5 modulates the rate of vascular development through violet-light exposure (Nguyen et al., 2019). Opn5 also allows for the photoentrainment of local circadian clocks in the murine retina, skin, and cornea (Buhr et al., 2015; Buhr et al., 2019). Deletion of *Opn5* in mice leads to a slower behavioral re-entrainment to shifted, “jet lag” light cycles (Ota et al., 2018).

The majority of mammalian cells express autonomous molecular circadian clocks (Yi et al., 2021). Interestingly, many neuronal populations throughout the brain are unique in that they do not show sustained autonomous molecular rhythmicity (Abe et al., 2002; Kriegsfeld et al., 2003). Notable exceptions to this include sustained rhythms in isolated SCN (the central timekeeper for the entire body) (Groos and Hendriks, 1982; Shibata et al., 1982; Shibata and Moore, 1988), arcuate nucleus (Abe et al., 2002), olfactory bulb (Granados-Fuentes et al., 2004), and hippocampus (Wang et al., 2009), among a few others. Here, we analyze the sustained rhythmicity and phase resetting properties of the VMPO. Many peripheral (non-SCN) oscillators can use temperature as a synchronizing time cue (Brown et al., 2002; Buhr et al., 2010). Similarly, the local circadian clocks of tissues which express Opn5 (retina, cornea, exposed skin) are directly photoentrainable (Buhr et al., 2015; Buhr et al., 2019; Díaz et al., 2020). We wished to analyze the ability of light and temperature to shift the local clocks of the VMPO, and to address any role for Opn5. We find that the cells with the strongest autonomous rhythmicity are medial to the Opn5-expressing cells, are not light sensitive, and are shifted by physiological temperature changes in an Opn5-independent manner.

## Materials and Methods

### Animals

All mouse procedures were carried out in accordance with regulations of the University of Washington Institutional Animal Care and Use Committee, Seattle, WA. All mice were of the background strain C57Bl/6J. Both male and female adult mice were used between the ages of 1 month and 1 year of age. *Opn5* ^*Cre*^ *; Ai14* mice were originally described in (Nguyen et al., 2019). These were crossed to *Per2* ^*Luciferase*^ mice (Jax strain # 006852) as described in (Yoo et al., 2004). *Opn5* ^*−/−*^ *; Per2* ^*Luciferase*^ are as described in (Buhr et al., 2015). *Opn5* ^*Cre*^ mice were also crossed to *Bmal1* ^*flox*^ mice (Jax strain # 007668) as described in (Storch et al., 2007).

### Lighting and Husbandry

Mice were weaned at 3 weeks of age and transferred from standard laboratory housing with white fluorescent lighting to housing cabinets with LED lighting which included violet (415-nm), blue (475-nm) and green (525-nm) light to activate known mouse retinal photoreceptors at a total intensity of 5 W/m^2^. Mice were exposed to a 12-h light: 12-h dark cycle. Mice were removed from the light cycle approximately 6 h into the light phase (*zeitgeber* time 18, “ZT 18”) and were euthanized with CO_2_ asphyxiation in room light for *ex vivo* luminescence experiments.

### Fluorescence Imaging

**FIGURE 1:**
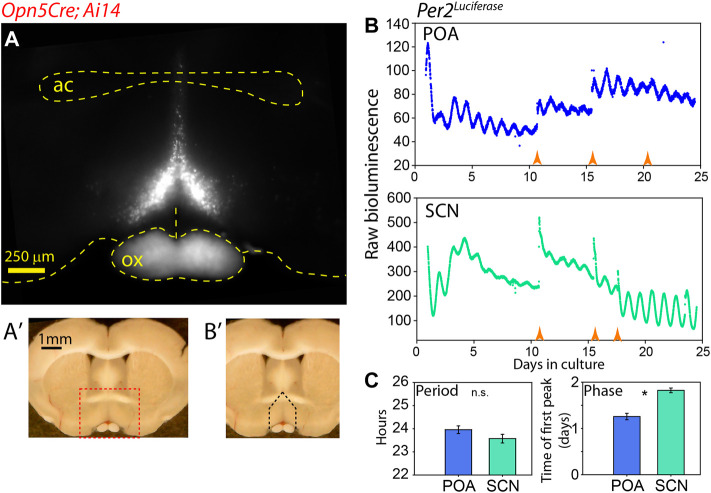
Autonomous clock region near Opn5 expression in POA. **(A)** Fluorescence image of tdTomato expression in coronal section of *Opn5* ^*Cre/+*^ *; Ai14* mouse. “ac” = anterior commisure, “ox” = optic chiasm. **(A**′**)** Whole brain slice showing region of detail in A as red dashed box. **(B**′**)** Same image as **(A**′**)** showing area of dissection for PMT measurement as black dashed lines. **(B)** Bioluminescence of *Per2* ^*Luciferase*^ from a coronal POA region as highlighted in **(B**′**)** (upper), and from the SCN of the same mouse (lower). Orange arrow indicate times of media change. **(C)** Free-running period (left) of and peak time of first full oscillation of *Per2* ^*Luciferase*^ bioluminescence POA and SCN from the same mice. Mean ± SEM are shown for *n* = 8. * = *p* < 0.05 paired *t*-test.

**FIGURE 2:**
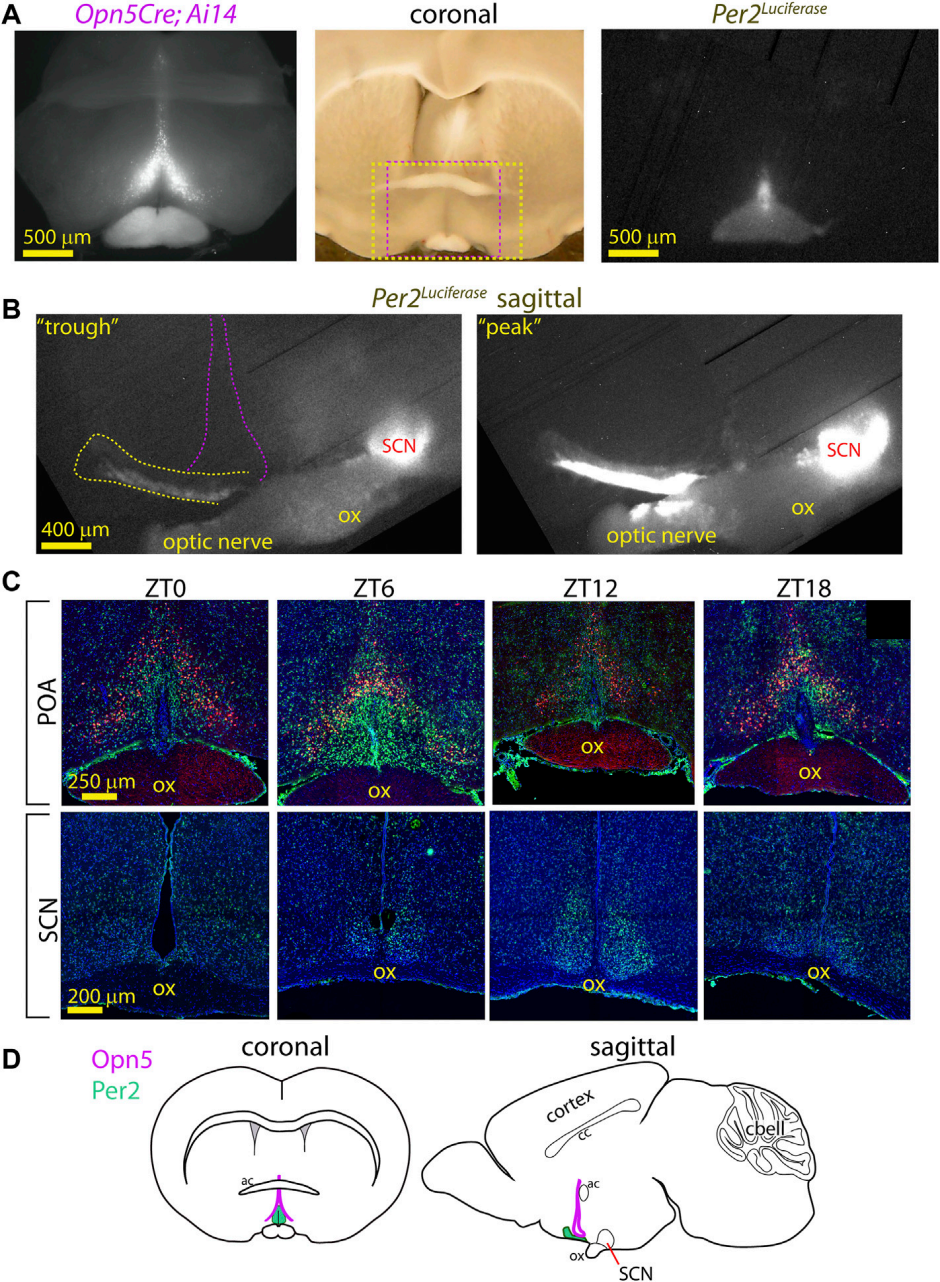
Anatomy of PER2 region near Opn5 expression in VMPO. **(A)** Fluorescence image of tdTomato expression in coronal section of *Opn5* ^*Cre/+*^ *; Ai14* mouse (left). Long-exposure (3 h) photo of *Per2* ^*Luciferase*^ bioluminescence from the same section (right). Center: Full light photo of coronal brain section with red dashed box indicating region of fluorescence (left) image and yellow dashed box indicating region of luciferase (right) image. **(B)** Bioluminescence of *Per2* ^*Luciferase*^ from a sagittal hypothalamus showing POA region (yellow dash) in reference to the SCN and the general area of Opn5 expression (violet dashed lines). 3 h exposures from a phase roughly centered on previous CT0 (left, “trough”) or CT12 (right, “peak”). “ox” = optic chiasm. **(C)** Fluorescence images of tdTomato (red), PER2 (green) and nuclei (blue) in POA (upper) and SCN (lower) in coronal slices of *Opn5* ^*Cre/+*^ *; Ai14* mice at the indicated *zeitgeber* times. *N* = 3 at each time point. **(D)** Schematic diagrams of the mouse brain with regions of Opn5 and POA-PER2 expression highlighted.

For fresh slices (Figures 1A, 2A), mice were euthanized with CO_2_ asphyxiation at ZT18 and brains were rapidly dissected in ice cold HBSS. Brains were sliced either coronally or sagittally with a vibroslicer (World Precision Instruments) and imaged on an Olympus IX81 microscope.

For immunohistochemistry, mice were exposed to 12-h light:12-h dark cycles of violet (415-nm), blue (475-nm) and green (525-nm) light for 2 weeks. At the indicated ZT phases (with ZT0 being times of lights-on and ZT12, lights-off) three mice per time point were euthanized by CO_2_ asphyxiation in dim red light and brains were rapidly removed into 4% paraformaldehyde. The brains were postfixed for 24 h, and cryoprotected in 30% sucrose in PBS. Brains were sectioned in the coronal plane at 20 μm sections in a cryostat (Leica CM 1850; Nussloch, Germany) at −20°C. Slices were incubated for 1 h in blocking buffer (2.5% normal goat serum, 2.5% normal donkey serum, 2% gelatin, 1% BSA, 0.2% Triton X-100) and then incubated for 1 h in 5% goat serum in PB + 0.3 % Triton X-100. They were then incubated in rabbit anti-Per2 primary antibody (PER21-A, Alpha Diagnostic Intl. Inc, San Antonio, TX, United States) diluted in PB + 0.3% Triton X-100 for 24 h. After incubation, samples were rinsed in PBS, incubated with secondary antibody goat anti-rabbit–Alexa 633 (Invitrogen, United States) for 1 h at room temperature, and mounted with ProLong Gold Antifade Mountant with 4′, 6-diamidino-2-phenylindole (DAPI) (Invitrogen). Immunofluorescent images were analyzed using a Leica DM6000 microscope with a Leica SP8 confocal system and processed with FIJI/ImageJ (National Institutes of Health, Bethesda, MD, United States). A separate researcher collected the tissues than analyzed the images who was blinded to the groups.

### Luminescence Imaging

Fresh 300 μm brain slices were placed on cell culture membranes (Millipore #PICMORG50) in DMEM (Cellgro) supplemented with B-27 Plus (Life Technologies), 2 mM Glutamax (Gibco), 10 mM HEPES buffer (Life Technologies), 25 U/ml penicillin; 25 μg/ml streptomycin (Sigma Millipore), 352.5 μg/ml NaHCO_3_, and 0.1 mM D-Luciferin potassuim salt (Biosynth). Cultures were maintained in 1.2 ml of media in 35 mm petri dishes which were sealed with sterile vacuum grease. Sealed dishes were maintained at 36°C with a microscope stage warmer and imaged with a Retiga Lumo camera (Teledyne Photometrics). Images of bioluminescence were collected over 1 h exposures and 3 1-h images were averaged into 1 using FIJI/ImageJ software. This procedure was repeated 4 times for each orientation (coronal or sagittal) of imaging.

### Luminescence Trace Measurements

Fresh 300 μm brain slices were cultured as above. Dishes were maintained in a Lumicycle PMT apparatus (Actimetrics) contained within an air-jacketed incubator (Sanyo MIR-262) at 36°C. POA and SCN were isolated from each mouse for *n* = 8. POA slices were approximately 600 μm anterior to the SCN. Raw data was used to measure periods using LM-Fit which uses a best-fit sine harmonic regression which also accounts for natural damping of the trace. Fourier power spectrum transforms with a time window between 22 and 40 h were performed using a Blackman-Harris filter within Lumicycle Analysis software (Actimetrics). Both period and Fourier amplitude was determined on days 2–7 in culture. Initial culture phase was determined using background-subtracted luminescence traces fit with a 2-degree polynomial fit line using Lumicycle Analysis and was determined as the time of the peak of the first full oscillation. For phase shifts, a best-fit sine wave (using LM-Fit which also adjusts for oscillation damping) was fit to data before or after a pulse. Phase shifts were measured as observed phase subtracted from expected phase. Amplitude was restored in brain slices by replacing culture media in Figure 1B.

### Light and Temperature Changes Ex Vivo

Cultured POA slices were transferred using an insulated shuttle box to a separate incubator which either included violet LEDs (peak output 415 nm) at 2 × 10^14^ photons cm^−2^ s^−1^ or a heating block set to warm a sealed dish to a stable 38°C. POA-containing dishes remained either exposed to violet light or to a temperature bock (in darkness) for 1.5 h before being returned to the Lumicycle in darkness.

For light cycles a custom light: dark apparatus was used as described in (Buhr and Van Gelder, 2014). Briefly, a 24-h clock motor rotated a disc above two diametrically opposed culture dishes containing organotypically cultured POA. A window in the otherwise opaque disk allowed light to pass through to the culture dishes only when directly above the tissue. A 10 h light: 14 h dark cycle was administered for four full 24-h cycles, so that one POA received the oppositely phased cycle as another (designated 0° and 180°). The light consisted of 415-nm at 2 × 10^14^ photons cm^−2^ s^−1^. Following the light: dark cycles the tissues were returned to constant darkness inside a Lumicycle apparatus. Time of the first peak after return to darkness was measured as the phase of traces after an *ex vivo* light:dark cycle.

### Statistics

Statistics were run using SigmaPlot 11.0. Two-tailed paired t-tests were used to compare the initial phase and periods of POA to SCN from the same animals. One-way ANOVA tests were used to test for differences beyond the 95% confidence intervals of multiple groups in phase shifts and of periods/amplitudes of more than two groups as in analyses of *Bmal1* ^*flox*^ experiments. No data was excluded.

## Results

### An Autonomous Circadian Clock in the VMPO

In previous work, we have observed a correlation between tissues which express the atypical opsin Opn5 and sustained clock gene expression (Buhr et al., 2015; Buhr et al., 2019; Díaz et al., 2020). We wished to analyze the molecular circadian clock properties of the VMPO where Opn5 is expressed (Zhang et al., 2020). We identified the region of interest with *Opn5* ^*Cre/+*^ *; Ai14* mice using tdTomato fluorescence as a marker. There is a pyramidal nucleus of Opn5-cells, approximately 500 μm in width at its base, which converges dorsally to its termination location just above the anterior commissure (Figure 1A). Although this Cre line lineage-marks cells during development, lacZ expression in *Opn5* ^*LacZ*^ mice and *in situ* hybridization has confirmed expression in adult brains within this region (Zhang et al., 2020). Using an *Opn5* ^*Cre/+*^ *; Ai14; Per2* ^*Luciferase*^ mouse line, we measured PER2 bioluminescence rhythms of the Opn5-containing region of the VMPO and the SCN of the same mice (Figure 1B) (Yoo et al., 2004). The VMPO displayed robust circadian oscillations of *Per2* ^*Luciferase*^ bioluminescence which could be reinitiated with a replacement of culture media. The free-running period of this region was similar to tissue cultures of SCN from the same mice, with POA oscillating at 23.9 ± 0.16 and SCN with 23.6 ± 0.2 (Figure 1C, *p* > 0.05 two-tailed *t*-test). The initial phase of POA was advanced compared with SCN, with the peak of the first full cycle in culture occurring approximately 6 h earlier than the SCN. This indicates that there is an endogenous clock within the POA that is capable of oscillating independently of the SCN.

A limitation of the above studies using photomultiplier tubes (PMT) is that the precise location of the luminescence cannot be assessed. We next imaged the POA of *Opn5* ^*Cre/+*^ *; Ai14; Per2* ^*Luciferase*^ using both a fluorescence microscope and a cooled CCD camera for long-exposure luminescence imaging. From the same slices we observed the extent of *Opn5* ^*Cre*^ *; Ai14* expression (tdTomato) and *Per2* ^*Luciferase*^ bioluminescence and saw that the PER2 reporter was at the midline near the third ventricle, roughly within the bounds of the Opn5-cells (Figure 2A). By slicing brains of *Per2* ^*Luciferase*^ mice sagittally the circadian activity of the VMPO and SCN can be observed in the same brain slice. The posterior boundary of the VMPO clock lies about 800 μm anterior to the SCN and extends rostrally for about 1200 μm (Figure 2B). The VMPO clock shows strongest *Per2* ^*Luciferase*^ activity in its most ventral portion but has rhythmic luminescence observable approximately 200 μm into the hypothalamus.

To further understand the anatomy of the relation of PER2 expressing cells to Opn5 cells, we collected brains from *Opn5* ^*Cre/+*^ *; Ai14* mice in a 12 h: 12 h light: dark cycle at four time points and antibody labelled for PER2. Of the times tested, the highest expression of PER2 in the VMPO was observed at ZT 6, and PER2 expression was medial to Opn5 expressing cells. Using an arbitrary threshold of PER2 expression in individual cells, 44 ± 8% of the Opn5 cells stained with the PER2 antibody at ZT 6 (*n* = 3, Figure 2C). In summary, the VMPO clock is contained within the boundaries of Opn5 cells with overlap, and the PER2 expression stretches anteriorly towards the rostral boundary of the hypothalamus (Figure 2D).

### The Circadian Clock in the VMPO is Not Photoentrainable

**FIGURE 3:**
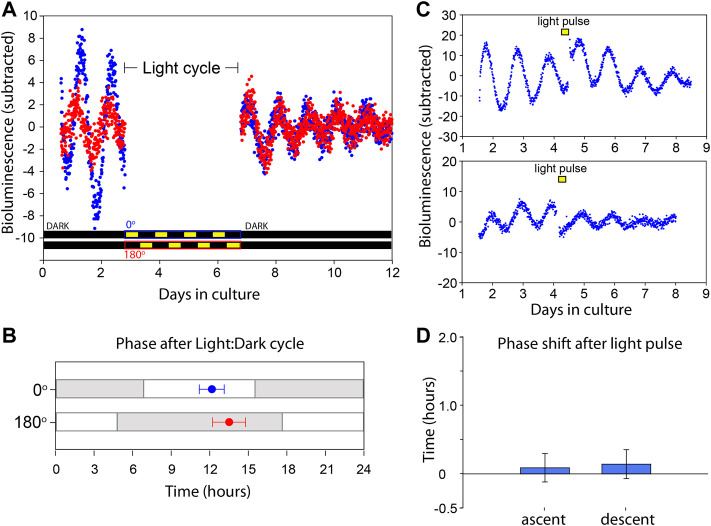
VMPO circadian clock is not directly entrained by light. **(A)** Bioluminescence traces of *Per2* ^*Luciferase*^ from two independent organotypic cultures (red and blue) of VMPO. The cultures are removed from PMT measurement and exposed to Light:Dark cycles from days 3 to 7. Light paradigms are indicated below for blue (0°) and red (180°) cultures. Light consisted of 415 nm, 2 × 10^14^ photons cm^−2^ s^−1^. **(B)** Phase of luminescence traces in constant darkness after exposure to oppositely phased Light:Dark cycles for 4 days. Points are average (±SEM) phases of first peak after return to PMT measurement with previous Light:Dark cycles indicated with gray and white shading. *n* = 8. **(C)** Bioluminescence traces of VMPO from *Per2* ^*Luciferase*^ mice given 90 min light pulses where indicated with a yellow box (415 nm, 2 × 10^14^ photons cm^−2^ s^−1^). **(D)** Average phase change (comparing phase of rhythm in days 5–7 to phase in days 1–4) after a light pulse administration occurring either in the early ascending phase of the luminescence rhythm (left, and upper panel of **(C)** or early descending phase (right, and lower panel of **(C)**. Shown are mean ± SEM. *n* = 5 each.

The photoentrainability of circadian clocks in close proximity to Opn5 expressing cells in other tissues inspired us to test for light sensitivity of circadian clocks in the POA (Buhr et al., 2015; Buhr et al., 2019). We made organoptypic cultures of POA from *Per2* ^*Luciferase*^ animals and placed them on opposite sides of a light apparatus that exposes two cultures to oppositely phased light: dark cycles of 415^ ^nm, 2 × 10^14^ photons cm^−2^ s^−1^ (Buhr and Van Gelder, 2014). After 4 days the tissues were returned to darkness and the bioluminescence phases were measured using PMTs. The phases of POA clocks after light: dark cycle exposure were the same as would be predicted by the phase prior to light exposure, and importantly, were not different between POAs receiving oppositely phased light: dark cycles (Figures 3A,B). In addition to light cycles (photoentrainment), we assessed the response to acute light pulses (phase shifts). After a light pulse of 415 nm, 2 × 10^14^ photons cm^−2^ s^−1^, for 90 min, the phase of POA rhythms were unchanged (Figures 3C,D). This was performed for phases both during the rising and descending phases of *Per2* ^*Luciferase*^ bioluminescence. Unlike the clocks in the retina, cornea, and skin (Buhr et al., 2015; Buhr et al., 2019; Díaz et al., 2020), the circadian clocks of the VMPO are not sensitive to violet light.

### The POA Clock can be Shifted by Temperature Changes, and This is not Mediated by Opn5 Cells

**FIGURE 4:**
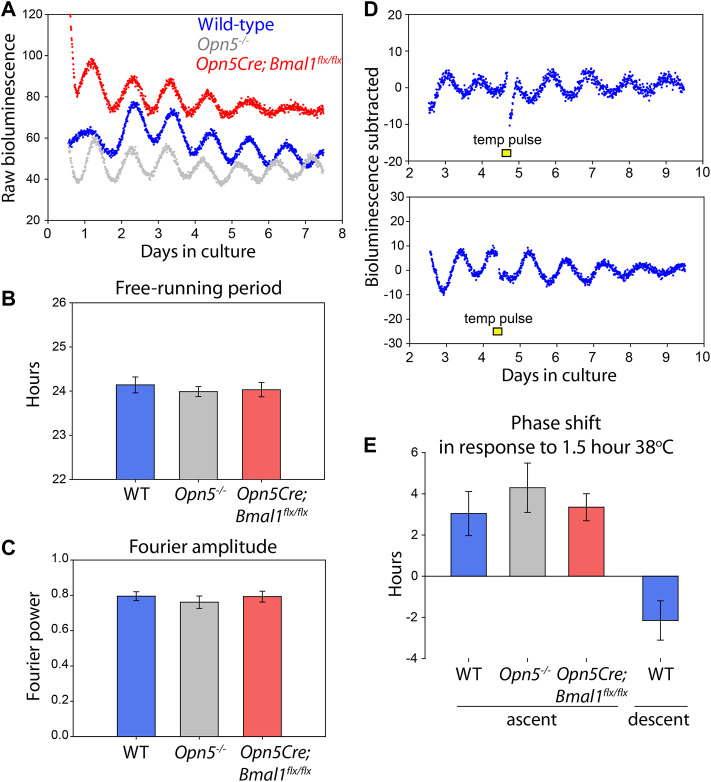
Opn5, and rhythms of Opn5-cells, are not necessary for VMPO rhythmicity. **(A)** Bioluminescence traces of *Per2* ^*Luciferase*^ from organotypic VMPO cultures from wild-type (*Per2* ^*Luciferase*^, blue), *Opn5* ^*−/−*^ *;Per2* ^*Luciferase*^ (gray), and Opn5-Bmal1-null (*Opn5* ^*Cre*^ *;Bmal1* ^*flx/flx*^ *;Per2* ^*Luciferase*^ *,* red) mice. **(B)** Free-running period of 5 days of oscillations as measured by LMFit (Lumicycle Analysis). **(C)** Fourier power-spectrum amplitude strength between 20 and 30 h with a Blackman-Harris filter of the same VMPO rhythms as **B** (Lumicycle Analysis). *n* = 8. Shown are mean ± SEM. ANOVA, *p* > 0.05. **(D)** Bioluminescence traces of VMPO from *Per2* ^*Luciferase*^ mice given 90 min 38°C temperature pulses (from a baseline of 36°C) where indicated with a yellow box. **(E)** Average phase change (comparing phase of rhythm in days 5–7 to phase in days 1–4) after a temperature pulse occurring either in the early ascending phase of the luminescence rhythm (left, three genotypes) or early descending phase (right). Shown are mean ± SEM. *n* = 8, 5, 6, and 5, respectively.

To further assess any influence of the POA’s Opn5 cells on the local circadian clock, we collected the POAs of *Opn5* ^*−/−*^ *; Per2* ^*Luciferase*^ and *Opn5* ^*Cre*^ *; Bmal1* ^*flx/flx*^ *; Per2* ^*Luciferase*^ mice. Compared to wild-type *Per2* ^*Luciferase*^ POAs, there were no differences in period or Fourier amplitude strength of mice lacking *Opn5* (*Opn5* ^*−/−*^) or lacking a functional clock within the Opn5-expressing cells (*Opn5* ^*Cre*^ *; Bmal1* ^*flx/flx*^) (Figures 4A–C). Neither the function of Opn5 itself nor the molecular circadian clock within the Opn5 cells directly modulates the free-running rhythms of the VMPO.

Because cells within this region of the POA are known to be temperature sensitive, we tested the ability of temperature changes within the physiologic range to shift the circadian clocks in the VMPO. Temperatures changes from 36 to 38°C for 1.5 h caused a stable change in the phase of the cultured VMPO tissue (Figure 4D). When given during the early rising phase of *Per2* ^*Luciferase*^ bioluminescence, a temperature pulse caused a 3.0 ± 1.1 h phase advance, and during the descending phase caused a −2.2 ± 0.9 h phase delay (Figure 4E).

Extra-ocular opsins have been implicated in thermal responses in mammalian spermatazoa (Pérez-Cerezales et al., 2015; Roy et al., 2020). To test if opsin-based thermal responses regulate the phase of the circadian clock, we also tested if the VMPO of *Opn5* ^*−/−*^ *; Per2* ^*Luciferase*^ and *Opn5* ^*Cre*^ *; Bmal1* ^*flx/flx*^ *; Per2* ^*Luciferase*^ mice responded normally to temperature pulses. We gave temperature pulses during the early rising phase of *Per2* ^*Luciferase*^ bioluminescence traces and measured the resulting phase shifts. *Opn5* ^*−/−*^ *; Per2* ^*Luciferase*^ VMPO shifted by 4.3 ± 1.2 h and *Opn5* ^*Cre*^ *; Bmal1* ^*flx/flx*^ *; Per2* ^*Luciferase*^ shifted 3.3 ± 0.6 h in response to 1.5 h temperature changes. This did not differ statistically from wild-type tissue (*p* > 0.05, one-way ANOVA). These results demonstrate that although Opn5 is expressed near to the circadian clocks of the VMPO, the presence of Opn5 itself and a functional clock within the Opn5-cells are not necessary for the free-running rhythms or the thermal responses of the VMPO circadian clock.

## Discussion

Here we describe an autonomous circadian clock in the most ventral aspect of the VMPO. This brain region displays robust rhythmic expression of the *Per2* ^*Luciferase*^ circadian reporter and can maintain oscillations for at least 1 month with fresh tissue culture media. These cells are near the third ventricle, but do not border it. Because this brain region also hosts the brain’s most concentrated expression of the atypical opsin, Opn5, we were interested in the direct photosensitivity of local VMPO circadian clocks (Zhang et al., 2020). In previous work we have observed that the presence of Opn5 is sufficient for the photoentrainment of surrounding tissue. In the retina, cornea, and pinna skin, Opn5 is expressed in a small subset of cells, but Opn5-dependent circadian photoentrainment is observed across diverse cell types throughout the local niche (Buhr et al., 2015; Buhr et al., 2019; Díaz et al., 2020). In the VMPO, Opn5 expressing cells straddle and overlap the areas in which we observed the strongest circadian rhythmicity. Thus we hypothesized that the violet light sensitive Opn5 cells may regulate the phase of the rhythmic VMPO cells. However, this was not the case. In both cyclic lighting conditions and acute light exposure, the phase of the circadian clocks in the VMPO were unchanged by direct light exposure.

A unique feature of the VMPO is the direct thermosensitivity of its warm-sensitive neurons (Nakayama et al., 1961) and the systemic response to local warming (Magoun et al., 1938). Because the brain is mostly insulated from environmental temperatures, changes in brain temperature occur mainly due to circadian variations, fever, or sleep. Our finding that the VMPO clocks are set by temperature changes within the physiologic range suggest that systemic body temperature may act as a synchronizing cue for the VMPO (Buhr et al., 2010). This is in addition to potential cues coming from the SCN *via* the subparaventricular zone (Vujovic et al., 2015). Many cells in the POA are activated by changes in environmental temperature as detected by cutaneous thermosensors (Nakamura and Morrison, 2008). Future work would be necessary to determine if this warm-induced activity also affects the local VMPO clock. This might present a discordant signal in a nocturnal animal for which environmental temperatures are typically cooler at night, when internal brain temperature experiences its maxima (Gao et al., 1995). It should be noted that neuronal firing is not required for temperature-based tissue entrainment of neural tissue, so the environmental temperature response may involve entirely separate mechanisms than clock phase alignment to internal temperatures (Buhr et al., 2010; Bedont et al., 2017).

A question arises as to why the VMPO contains autonomous circadian clocks, whereas so much of the surrounding hypothalamic tissue does not. The VMPO has numerous efferent targets, including the dorsomedial hypothalamus (DMH), arcuate nucleus, and the paraventricular nucleus (PVN), which support the VMPO’s role in autonomic modulation of body temperature (Tan et al., 2016). The fact that lesioning of the VMPO leads to a rhythm of core body temperature with a higher amplitude than normal suggests a potential role for the VMPO clock in rhythmically regulating temperature maxima and minima (Osborne and Refinetti, 1995; Lu et al., 2000). A future tracing study would be helpful to determine the specific output targets of the VMPO clock cells.

Finally, while this work demonstrates that these Opn5-expressing cells are not necessary for the circadian function or thermal entrainment of the VMPO clock, it was limited in determining if the Opn5-cells themselves express functional circadian clocks. While we did observe that ∼44% of *Opn5* ^*Cre*^ *; Ai14* cells co-expressed PER2 at peak expression, because PER2 is expressed as a gradient over time, this required use of an arbitrary threshold. It is possible that an underlying PER2 rhythm is present in Opn5-cells but is too low to be measured in the presence of the strongly rhythmic cells near the third ventricle (Figure 2). In the future, a Cre-based reporter system could address this directly. However, our results indicate that any *Bmal1*-based rhythms within the Opn5 cells are not directly related to the circadian function of the primary VMPO clock. The autonomous circadian clocks within the VMPO appear to be functionally isolated from the violet-light sensitive Opn5-cells which surround them.

## Data Availability

The original contributions presented in the study are included in the article/Supplementary Material, further inquiries can be directed to the corresponding author.
